# Monkeypox Virus Infection of Rhesus Macaques Induces Massive Expansion of Natural Killer Cells but Suppresses Natural Killer Cell Functions

**DOI:** 10.1371/journal.pone.0077804

**Published:** 2013-10-17

**Authors:** Haifeng Song, Nicole Josleyn, Krisztina Janosko, Jeff Skinner, R. Keith Reeves, Melanie Cohen, Catherine Jett, Reed Johnson, Joseph E. Blaney, Laura Bollinger, Gerald Jennings, Peter B. Jahrling

**Affiliations:** 1 Integrated Research Facility, National Institute of Allergy and Infectious Diseases, National Institutes of Health, Frederick, Maryland, United States of America; 2 Computational Biology Section, Bioinformatics and Computational Biosciences Branch, National Institute of Allergy and Infectious Diseases, National Institutes of Health , Bethesda, Maryland, United States of America; 3 New England Primate Research Center, Harvard Medical School, Southborough, Massachusetts, United States of America; 4 Emerging Viral Pathogens Section, National Institute of Allergy and Infectious Diseases, National Institutes of Health , Bethesda, Maryland, United States of America; Centers for Disease Control and Prevention, United States of America

## Abstract

Natural killer (NK) cells play critical roles in innate immunity and in bridging innate and adaptive immune responses against viral infection. However, the response of NK cells to monkeypox virus (MPXV) infection is not well characterized. In this intravenous challenge study of MPXV infection in rhesus macaques (*Macaca mulatta*), we analyzed blood and lymph node NK cell changes in absolute cell numbers, cell proliferation, chemokine receptor expression, and cellular functions. Our results showed that the absolute number of total NK cells in the blood increased in response to MPXV infection at a magnitude of 23-fold, manifested by increases in CD56+, CD16+, CD16-CD56- double negative, and CD16+CD56+ double positive NK cell subsets. Similarly, the frequency and NK cell numbers in the lymph nodes also largely increased with the total NK cell number increasing 46.1-fold. NK cells both in the blood and lymph nodes massively proliferated in response to MPXV infection as measured by Ki67 expression. Chemokine receptor analysis revealed reduced expression of CXCR3, CCR7, and CCR6 on NK cells at early time points (days 2 and 4 after virus inoculation), followed by an increased expression of CXCR3 and CCR5 at later time points (days 7-8) of infection. In addition, MPXV infection impaired NK cell degranulation and ablated secretion of interferon-γ and tumor necrosis factor-α. Our data suggest a dynamic model by which NK cells respond to MPXV infection of rhesus macaques. Upon virus infection, NK cells proliferated robustly, resulting in massive increases in NK cell numbers. However, the migrating capacity of NK cells to tissues at early time points might be reduced, and the functions of cytotoxicity and cytokine secretion were largely compromised. Collectively, the data may explain, at least partially, the pathogenesis of MPXV infection in rhesus macaques.

##  Introduction

 Monkeypox virus (MPXV), an emerging virus that could cause up to 10% lethality in humans, poses a risk to human health as an infectious agent and as a potential biological weapon. Human MPXV infection resembles, in many aspects, clinical symptoms of smallpox, including fever, weight loss, lesions, and death [1,2]. To fully understand the pathogenesis of MPXV and related orthopoxviruses, such as variola virus, and to provide a model to evaluate countermeasures to these poxviruses, a MPXV non-human primate (NHP) model has been developed [3,4]. NHPs infected with MPXV have symptoms resembling those of human monkeypox and smallpox. Intravenous (IV) injection of high doses (~5 x 10^7^ PFU) of MPXV led to 80% lethality in macaques and injection of 5 x 10^6^ PFU of MPXV resulted in survival of most macaques (>80%) [3,4]. While most studies were focused on the pathology and pathogenesis of MPXV, little is known about the innate and adaptive immune responses to MPXV infection in this model. 

 Natural killer (NK) cells are a major arm of innate immunity and play a critical role in anti-viral immune responses [5]. The activation or inhibition of NK cells is initiated by interaction between activating or inhibitory receptors on NK cells and their ligands, such as major histocompatibility complex I (MHC-I) molecules, on target cells [6,7]. NK cells exert their antiviral protective role by direct killing of virus-infected cells and by secreting cytokines to modulate functions of other cell types such as T cells and dendritic cells (DCs) [8-10]. The killing effect of NK cells is mediated by cell-cell interactions and by secretion of granules containing perforin and granzymes [11]. Cytokines, such as interferon gamma (IFN-γ) and tumor necrosis factor alpha (TNF-α), secreted by NK cells at early stages of infection mediate inflammatory responses in inflamed tissues and also coordinate with DCs to induce Th1 cell polarization [12,13]. In both animal models and in humans, NK cells are essential to controlling replication of some viruses, such as herpes viruses in humans [14], murine cytomegalovirus [15,16], human immunodeficiency virus (HIV)[17], influenza viruses [18], and ectromelia virus (ECTV) [19,20]. Depletion of NK cells in mice rendered mice more susceptible to a number of viruses, such as murine hepatitis virus, murine cytomegalovirus , and vaccinia virus (VACV), but had no effect on susceptibility of mice to lymphocytic choriomeningitis virus [21]. These data suggest that the relative importance of NK cells in controlling virus invasion may vary from virus to virus [22-24]. 

 Studies on NK cell function in orthopoxvirus infections have been primarily performed in mouse models. In the ECTV orthopox model, C57BL/6 mice are naturally resistant to ECTV via foot-pad inoculation. Previous data indicates that NK cells are essential for protecting C57BL/6 mice from ECTV by direct cytolytic function and by modulating T cell responses [20,25,26]. By depleting NK cells at different time points after ECTV infection, Fang and Sigal showed that NK cells are required at early stage of infection (at and before day 4) to control viral loads and confer resistance to ECTV infection [20]. Furthermore, aged C57BL/6 mice became susceptible to ECTV infection owing to reduced NK cell number and compromised trafficking of NK cells to lymph nodes (LNs) [27]. In addition to ECTV, NK cells are also essential to curb the replication of another orthopoxvirus, VACV [21-23]. However, the NK cells during ECTV and VACV infection may use different mechanisms to control replication of these viruses [22]. 

 In humans, CD56 is commonly used as a NK cell marker. However, CD56 is not specifically expressed on all NK cells, but on subsets of NK cells in rhesus macaques [28]. Instead, almost all NHP NK cells express CD8α and NKG2A, thus, CD3-CD8+NKG2A is used as a reliable combination of markers to identify NK cells in NHPs [29,30]. In addition, different subsets of NHP NK cells can be classified by CD16 and CD56 expression on the cell surface. While the CD16+CD56- subset (CD16+ NK cells) is the dominant subset (85-90%) in the blood at steady state, a low frequency of CD16-CD56+ (CD56+), CD16-CD56- (double negative, DN), and CD16+CD56+ (double positive, DP) NK cells also exist in circulating blood [30,31]. Transcriptional analysis suggested that CD16+, CD56+, and DN cells from rhesus macaques are distinct, but inter-related NK cell subsets with each population having unique, but also overlapping expression profiles such as chemokine and cytokine receptors and transcriptional factors [32]. Similar to human NK cells [33], NHP CD16+ NK cells primarily exert cytotoxic function, and CD56+ cells are prone to secrete cytokines. The DN cells have functions of both cytotoxicity and secretion of cytokines [31,33,34]. Previous studies in macaques have assessed NK cell numbers and/or functions following challenge with other viruses. Peripheral NK cell number decreased about 2-fold at 3-4 days post-inoculation when rhesus macaques were infected with lymphocytic choriomeningitis virus WE strain [35]. In studies with Ebola and Marburg viruses in macaques, both viruses also induced depletion of NK cells in the blood [36,37]. In studies with simian immunodeficiency virus infection (SIV), acute infection of NHPs with SIVmac251 resulted in activation and increased lytic capacity of NK cells that appeared to be associated with control of SIV infection [38]. In contrast, rhesus macaques chronically infected with SIV displayed impaired cytokine expression, but surface CD107a expression did not change compared to uninfected NHPs [31]. 

 In the current study, we assessed for the first time the changes in NK cell numbers and phenotype in the blood and lymphoid tissues of NHPs upon sub-lethal MPXV IV inoculation. We also analyzed NK cell proliferation, the expression patterns of cell surface chemokine receptor, and NK cell functions. Our data indicate that MPXV infection induced marked increases in NK cell numbers and cell proliferation. However, the capacity of NK cells to migrate to peripheral tissues might be reduced at early time points following virus inoculation, as reflected by reduced chemokine receptor expression on the surface of NK cells. Most importantly, NK cells both in the blood and in the lymphoid tissues displayed a compromised degranulation capacity and lost almost all cytokine secreting potential. 

##  Materials and Methods

### Ethics statement

All animal experiments were approved by the National Institute of Allergy and Infectious Diseases, Division of Intramural Research, Animal Care and Use Committee (ACUC) and adhered to National Institutes of Health (NIH) policies. The entire procedure to handle NHPs including injections or inoculations of virus, blood withdrawals, and biopsy and necropsy strictly followed NIH NHP regulations and guidelines. All animal handling and scientific procedures (e.g. virus injection, blood withdrawal, and biopsy) were performed under anesthesia with intramuscular injection of ketamine hydrochloride 10-25mg/kg. Investigators and animal care personnel provided additional comfort and care such as supplemental heat and IV, SQ, or oral fluids, consistent with the scientific integrity of the protocol. Animals that were not eating were tube-fed under sedation. Such animals were given metoclopramide 0.3 mg/kg IM 15 minutes prior to tube feeding to prevent vomiting.

Animals were euthanized in accordance with the 2007 Report of The American Veterinary Medical Association Panel on Euthanasia utilizing exsanguination via direct cardiac puncture subsequent to the induction of deep anesthesia by intravenous injection of 100 mg/kg of sodium pentobarbital (Fatal Plus®, Vortech Pharmaceuticals, Dearborn, MI). Death was ensured by administration of sodium pentobarbital.

### Animals, Virus Inoculation, and Sample Collection

 MPXV Zaire 79 stocks were propagated in BSC-1 cells at a multiplicity of infection (MOI) of 0.1 for 3 days. Virus inoculum was crude virus prepared by 3 freeze/thawing cycles followed by centrifugation and ultrasonic treatment, then pelleted over a 36% sucrose cushion. 

The experimental design is outlined in Table 1. Rhesus macaques (*Macaca mulatta*) of both sexes, ranging in weight from 4–8 kg, were housed in biosafety level 3 (BSL-3) bio-containment cages (Primate Products, Miami, FL). Such macaques were acclimated to a 12:12 hour light/dark cycle in a temperature and humidity controlled vivarium facility at NIH. All animals were acclimated to the study facility for a minimum of 2 weeks prior to the start of the study. Prior to enrollment, NHPs were screened and found to be seronegative for simian retrovirus, simian T cell leukemia, VACV, cowpox virus, and MPXV. Virus inoculations were performed by IV injection of desired virus doses in 1ml of phosphate buffered saline (PBS). At different time points before and after virus inoculation, blood was collected aseptically in ethylenediaminetetra-acetic acid (EDTA)-treated tubes. Blood samples were tested for changes in standard clinical serum chemistry values, virus load, and total cell counts of blood cell populations to monitor subject health. Clinical signs were evaluated daily. Biopsies of axillary LNs at day 5 and day 12 after virus inoculation in Experiment B and complete necropsies at the end of each experiment were performed following standard surgical procedures.

**Table 1:** Overview of experimental MPXV IV challenge protocols for experiments A and B.

Experiment	MPXV Virus Strain	Inoculation (IV) dose (PFU^a^)	No. of NHPs	Blood samples (days)	Lymph node biopsy (days)	Necropsy study endpoints (days)
**A**	Zaire 79	2.5 x 10^6^	6	0, 2, 4,7, 8, 9	no	8, 9
**A**	Normal control	0	2	0, 2, 4, 7, 8, 9	no	8, 9
**B**	Zaire 79	1.5 x 10^6^	5	0, 2, 5, 8, 12, 16, 21, 30	5, 12	30
**B**	Normal control	0	2	0, 2, 5, 8, 12, 16, 21, 30	5, 12	30

PFU = plaque-forming units

 The data reported herein are results from two individual experiments. Experiment A included six MPXV-infected NHPs (2.5 x 10^6^ PFU Zaire 79 strain) and two normal controls. We performed the following analyses on samples from experiment A: 1), chemokine receptor staining from peripheral blood mononuclear cells (PBMCs) at different time points and LN cells from days 8 and 9 necropsy samples; 2), NK cell frequency and total NK cell number from whole blood and day8/9 LNs; and 3), NK cell functional analysis (CD107a, IFN-γ, TNF-α) from PBMCs and LN cell samples. In experiment B, five NHPs were infected with 1.5 x 10^6^ MPXV Zaire 79 strain and another two NHPs served as uninfected controls. We obtained the data for the total NK cell number in the blood and the Ki67+ expression data in the blood and LNs (days 5 and 12) from Experiment B. 

### Reagents and antibodies

 The following antibodies (Abs) and conjugates were purchased from BD Pharmingen (San Jose, CA): CD3-Pacific Blue, -Alexa Fluor^®^ 700, -APC (SP34-2); CD8α-APC-H7 (SK1); CD8α-Pacific Blue (custom conjugate, SK1); CD14-Pacific Blue, -Alexa Fluor^®^ 488 (M5E2); CD16-APC, -PE (3G8); CD45-PerCP (D058-1283); CD56-PE-Cy7 (NCAM16.2); CD197 (CCR7)-Alexa Fluor^®^ 647; CD183 (CXCR3)-PerCP-Cy5.5 (1C6); CD195 (CCR5)-APC (3A9); CD196 (CCR6)-PerCP-Cy5.5 (11A9); human Ki67-PerCP-Cy5.5 (B56); CD107a-APC (H4A3); INFγ-PerCP-Cy5.5 (B27); TNFα-V450 (Mab11); mouse IgG2a-AlexaFluor^®^ 647, -APC (G155-178); and mouse IgG1-PerCP-Cy5.5 (MOPC-21). CD16-FITC (VEP13) was purchased from Miltenyi Biotech (Auburn, CA). CD159a (NKG2A)-PE (Z199) was obtained from Beckman Coulter (Miami, FL). Yellow LIVE/DEAD^®^ dye was purchased from Invitrogen (Carlsbad, CA).

### Preparation of cells from blood and LNs

 EDTA blood was either treated with ammonium-chloride (ACK) lysis buffer to obtain leukocytes or layered over Ficoll density gradient to obtain PBMCs. To obtain leukocytes, 0.7 ml of whole blood was suspended in 3 mL of ACK buffer (Invitrogen, Carlsbad CA) for 2–3 min, centrifuged, and washed twice with PBS containing 2% fetal bovine serum (FBS). For the preparation of PBMCs, EDTA-blood was diluted 2-fold in PBS, layered over Ficoll solution (Sigma-Aldrich, St. Louis, MO), and centrifuged at 1000 x g at room temperature (RT) for 30 min. Then, the interface cell layer was collected and washed twice with PBS+2% FBS. The PBMCs were either directly used for flow cytometric staining or for NK cell functional assays.

To prepare cells from LNs, the axillary LN was placed in a cell strainer in a petri-dish containing 10 mL of PBS with 2% FBS. The tissues were gently ground with the tip of a syringe. The cells were collected and washed twice with PBS containing 2% FBS for further analysis.

### Surface and intracellular staining

 To characterize NK cells and NK subsets, PBMCs or leukocytes (0.5–1 x 10^6^ cells) were incubated with a mixture of antibodies (Abs) to cell surface markers for 15–20 min at RT. For chemokine receptor staining, PBMCs were incubated at 37°C in RPMI-1640 medium containing 5% FBS for one hours followed by staining of cells with Abs against chemokine receptors at 37°C for one hour. Cells were then washed and subsequently stained with antibodies against CD3, CD8, CD16, CD56, and NKG2A for 30 min at RT. Then the cells were washed with PBS+2% FBS and were suspended in BD FACS lysing buffer (BD Biosciences, San Jose, CA) for 30–40 min to inactivate potential viruses before data acquisition. For intracellular staining, the cells were washed twice with PBS+2% FBS, then fixed with Cytofix/Cytoperm (BD Biosciences) for 30–40 min at RT. The cells were washed with BD Perm/Wash, followed by incubation with Abs to Ki-67 or cytokines. After washing with BD Perm/Wash, the cells were suspended in Perm/Wash buffer and were acquired using a LSR-Fortessa cytometer (Becton Dickinson, San Jose, CA). The data were analyzed with Flowjo software (TreeStar Inc., Ashland, OR) or with BD FACS Diva software (BD Biosciences).

### Absolute NK cell counts in blood and axillary LNs

 The absolute number of NK cells in the blood was obtained in two steps. First, in BD Trucount^™^ tubes (BD Biosciences), the absolute number of blood CD45+ leukocytes, CD3+, CD8+ and CD4+ T cells and B cells were obtained. In a second set of tubes, blood cells were stained with markers to distinguish NK cells and subsets including CD3, CD8, NKG2A, CD16, and CD56. The absolute number of CD16+, DN, CD56+, and DP subsets within the NKG2A gate was calculated based on the absolute number of CD3-CD8+ population from the Trucount tubes. 

The absolute number of total live cells in each axillary LN was obtained by Trypan Blue exclusion, and the frequency of NKG2A+ NK cells within the lymphocyte gate was obtained by flow cytometric staining. Then the total NK cells (NKG2A+) and NK subset cell number were calculated based on the total LN cell number and the frequency of NK cells and NK subsets in the LNs. 

### NK cell functional assay

 To analyze NK cell function, we adapted a method published by Reeves et al. [31]. NK cells were enriched by depleting CD3+ T cells with CD3+ T cell microbeads (Miltenyi Biotech Auburn, CA). The resulting cell population contained less than 3% of CD3+ T cells. NK-enriched cells (1 x 10^6^ cells) were co-cultured with MHC-I negative human cell line 721.221 at a ratio of 5:1 for 10–12 h at 37°C in 5% CO_2_ in serum-free medium, Aim-V^®^ (Life Technologies, Grand Island, NY). Cultures containing phorbol myristate acetate (50 ng/mL) and ionomycin (200 ng/mL) or medium alone served as positive and negative controls, respectively. Anti–CD107a-APC (clone H4A3) was added into the culture (1μL/mL) during the incubation period and BD Golgiplug and Golgistop (BD Biosciences) were added into the culture as manufacturer recommends. Following overnight culturing, the samples were stained using surface markers to reveal NK cells and NK subsets (CD3, CD8, NKG2A, CD16, and CD56). The cells were then fixed and permeabilized using BD Cytofix/cytoperm followed by intracellular cytokine staining with Abs against IFN-γ and TNF-α.

### Quantification of viral load in the whole blood by qPCR

 Viral load in whole blood was determined by quantitative PCR (qPCR) after DNA isolation from 50 µl of whole blood using Genfind v2 according to the manufacturer’s directions (Agencourt, Danvers, MA) as described previously [39]. Results were calculated by reference to the standard curve, multiplied by the appropriate dilution factor, and reported as gene copies per ml. The lower limit of detection of the synthetic oligo was 10 copies, while the upper limit was 1x10^8^ gene copies per ml. We set our limit of detection at 10,000 copies/ml blood so that we were well within the standard deviations that would account for dilution errors and extraction efficiencies.

### Statistical analyses

 Statistical and graphic analyses were completed using GraphPad Prism software (GraphPad Software Inc, San Diego, CA) and the R Project for statistical computing (R Core Team, Vienna Austria). Most experiments were tested using repeated measure two-way ANOVA with Bonferroni post-tests. Student t-tests were also used in some analysis. *P* values less than 0.05 (*p*<0.05) were assumed to be significant in all analyses. All analyses were verified using appropriate bootstrap and permutation re-sampling tests [40,41] in situations where the assumptions were not met for parametric T-tests and ANOVA or for analogous nonparametric Mann-Whitney and Kruskal-Wallis tests. Wherever needed, Bonferroni or Holm adjustments for multiple tests were used. Statistical analysis for the correlation between virus load and NK cell number was using both Pearson correlation and Spearman rank correlation test.

##  Results

### Marked increases in peripheral blood NK cell frequency and absolute numbers following MPXV challenge

**Figure 1:**
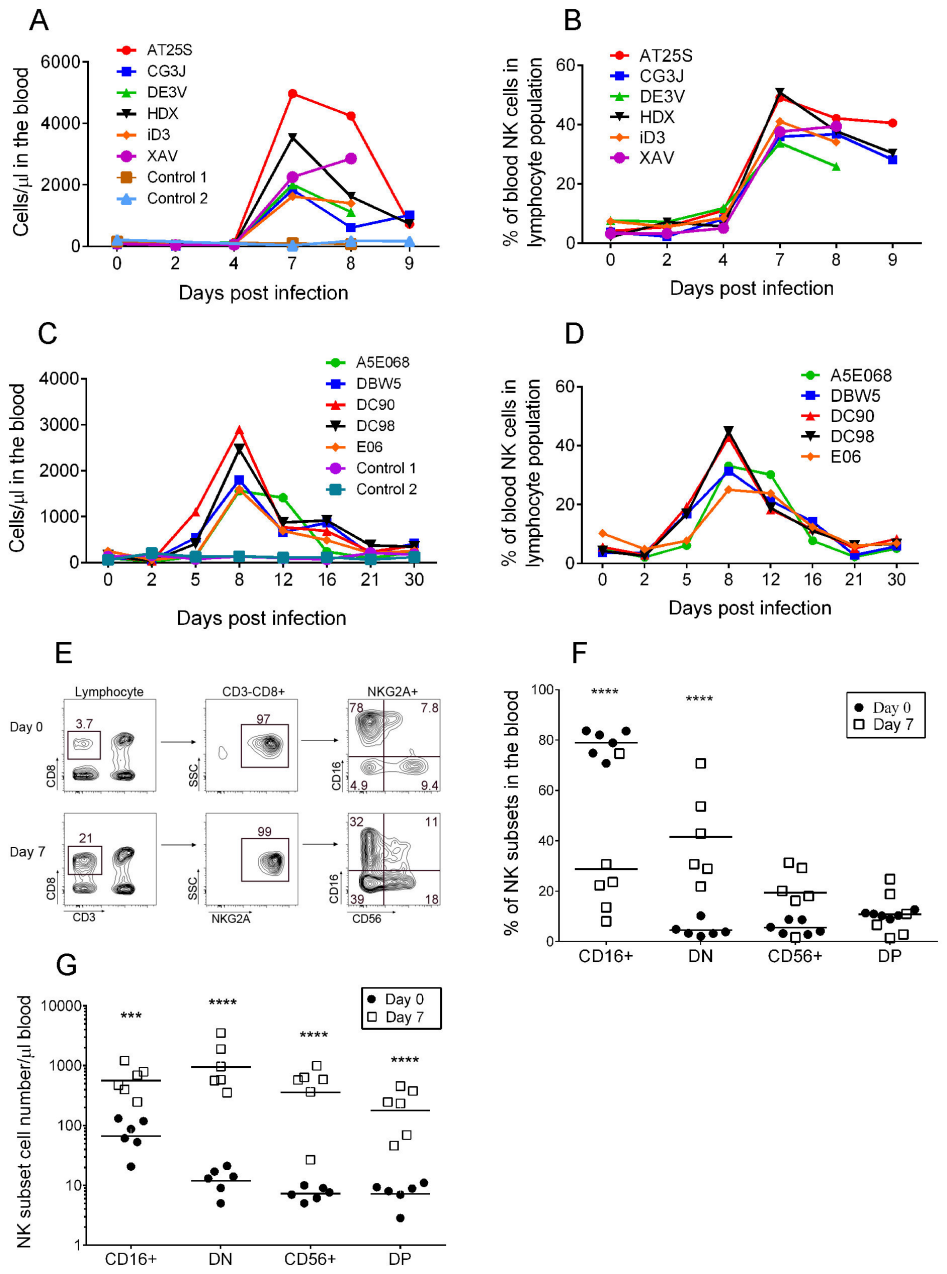
MPXV infection induced increases in NK cell frequency and absolute number in the blood. Kinetics of NK cell total number (A, C) and frequency (B, D) in the blood from experiments A (A, B) and B (C, D), respectively. E) Gating strategy of NK cell subsets from day 0 and day 7 after virus inoculation. F) Frequency of NK cell subsets within the total NK cell gate in the blood at day 7 after virus inoculation (bar represents the median frequency). G) Absolute number of NK cell subsets in the blood at day 7 (bar represents the median NK cell number/µl of blood). Statistical analysis of F) and G) were computed using repeated measures two-way ANOVA in Graph-pad Prism software with Bonferroni post-tests and only significant *p* values were shown (***p<0.001; and ****p<0.0001). Results were confirmed using bootstrap re-sampling test computed with the R project for statistical computing.

 Previous studies with MPXV challenge in NHPs reported that MPXV infection induced lymphoid depletion and lymphadenopathy [3,4]. This raises the question whether MPXV infection induces changes in the number of lymphocytes including NK cells in NHPs. From blood samples collected at different time points (Table 1), the absolute numbers of lymphocytes and NKG2A+ NK cells or CD3-CD8+ NK-enriched cells were enumerated. The number of lymphocytes slightly decreased from baseline to day 2 and day 4 and then increased on average 3.0-fold at day 7 in experiment A (Figure S1A) and 2.1-fold at day 8 in experiment B (Figure S1B). After day 8, overall lymphocyte counts remained high in a range of 1.5-2-fold of the base line (Figure S1B). The baseline number of NK cells in rhesus macaques from our study was 116 cells/μl of blood (range from 26–232 cells/μl blood) (Figure 1A, Experiment A). At days 2 and 4 after MPXV inoculation, the absolute numbers of NK cells remained relatively unchanged. NK cell counts began to increase at around day 5, and peaked (average 2704 cells/μl blood) at day 7 after virus inoculation, representing an average increases of 23-fold over the baseline counts. At day 8, the NK cell number started to decline, but was still 17-fold higher than the baseline (Figure 1A). Accordingly, the frequency of NK cells of the total lymphocyte population rose from an average 4.7±2.3% at baseline to 41.4±7.0% at day 7 and 36.0±5.6% at day 8 (Figure 1B, Experiment A). Results from experiment B confirmed the kinetics of NK cell numbers with a peak at day 8, representing an average increase from 5.7±2.6% of the total lymphocyte population at baseline to 35.4 ±8.2% at day 8, followed by a decline close to baseline at day 21 (Figure 1C, 1D). The changes in NK cell numbers upon MPXV infection varied markedly among individual NHPs. For example, NHP AT25S showed a 48-fold increase (4969 cells/μl blood at day 7 versus 104 cells/μl blood at baseline), while NHP iD3 had only a 10-fold increase (1627 versus157 NK cells/μl blood at day 7 or day 0, respectively) (Figure 1A). 

We questioned if all NK subsets or specific subset(s) increased in the blood following MPXV challenge. NK cell subsets were distinguished based on CD16 and CD56 expression within the NKG2A+ NK cell gate (Figure 1E) [31]. The frequency of CD16+ NK subset within the total NK cell population decreased from 78.7 ± 4.5% at day 0 to 28.8 ± 21.7% (p< 0.0001 ) at day 7 post MPXV inoculation (Figure 1F). The frequency of DN cells increased from 4.6 ± 2.7% to 41.4 ± 16.7% (*p*<0.0001), becoming the dominant NK cell subset in the blood. In addition, CD56+ NK cells also increased from 5.5 ± 2.4% to 19.4 ± 9.7% (p=0.18) and DP NK cell frequency remained unchanged (10.7 ± 1.2% and 10.9 ± 8.5%, p>0.05). The absolute number of all NK subsets at day 7 postinoculation significantly increased (p<0.001 or p<0.0001) upon MPXV infection (Figure 1G). Among them, the DN population showed maximal increase (around 100-fold). For CD16+, CD56+, and DP NK cells, the increases were 8.1-, 71-, and 30-fold on average (Figure 1G). 

### Increased NK Cell Numbers in Lymphoid Tissues during MPXV Infection

**Figure 2:**
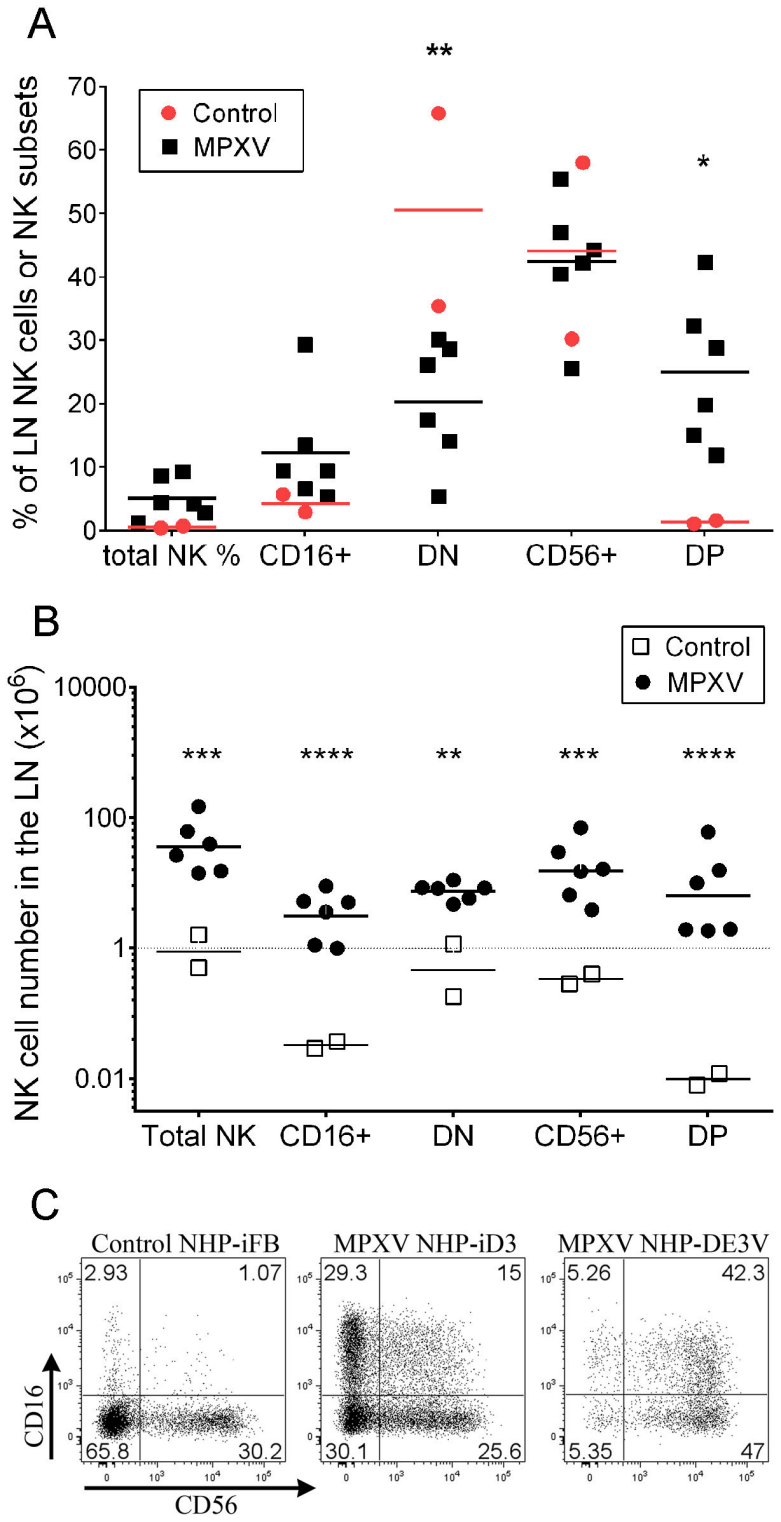
NK cell frequency and absolute number in LNs at days 8–9 after MPXV inoculation. A) Frequency of total NK cells in the LN lymphocyte gate and frequency of NK subsets in the NK cell gate. B) Total NK cell number and numbers of NK subsets in the LN. Total NK cell number was calculated based on the frequency of NKG2A+ NK cells in the lymphocyte gate and the total live cell number of individual LNs. The total number of NK cell subsets was calculated based on the frequency of each subset within the NK cell gate and the total NK cell number in the LN. C) Dot-plot showing the marked variation of NK cell subset distribution in two representative subjects induced by MPXV infection. Statistical analyses of A) and B) were computed using two-way ANOVA in Graph-pad Prism software with Bonferroni post-tests and *p* values with significant change are shown (* p<0.05, **p<0.01, *** p<0.001, **** p<0.0001). Results were confirmed using bootstrap re-sampling test computed with the R project for statistical computing.

 We questioned if MPXV infection induced changes in NK cell number and composition in the LNs. Our analysis showed that the frequency of total NK cells among lymphocytes increased about 8.4-fold from an average of 0.55% in control NHPs to 4.6% in MPXV-infected NHPs (Figure 2A). Accordingly, the total number of NK cells per axillary LN dramatically increased from an average of 1.1x10^6^ cells in control NHPs to 50.7 x10^6^ cells in MPXV-infected NHPs (Figure 2B). The magnitude of change in NK cell numbers varied greatly among individual MPXV-infected NHPs. For example, the total NK cell number was 14.1 x10^6^ per LN in one NHP and it was 147.5 x10^6^ in another NHP (Figure 2B). 

 Analysis of NK cell subsets in the axillary LNs from control NHPs confirmed previous reports [31,42] that DN and CD56+ cells were the major NK cell subpopulations in the LNs of uninfected NHPs (Figure 2A, B and C). At days 8–9 post-inoculation, the CD16+ and DP NK cells in the LNs increased at variable frequencies. For example in NHP iD3 (Figure 2C), CD16+ population reached 29.3% of total NK cells and DP cells accounted for 15% of total NK cells. However, in NHP DE3V, the CD16+ and DP populations accounted for 5.26% and 42.3% respectively, of the total NK cells. Across all MPXV-infected NHPs, the change in CD16+ cell frequency was not significant (p>0.05). DN cell frequency was reduced from average of 50.6% in control NHPs to 20.3% in MPXV-infected NHPs (p<0.01). CD56+ cell frequency remained unchanged (p>0.05). Regardless the increase or decrease in cell frequency, the absolute number of all subsets significantly increased as a result of the marked increase of total NK cell number in the LNs (p<0.01, p<0.001, or p<0.0001) (Figure 2B). Of the four NK cell subsets, DP cells on average displayed the greatest magnitude of increase. This increase in DP cells is partially a consequence of the extremely high number of total NK cells (147.5 x 10^6^) and DP cells (60.1 x 10^6^, 42.3% of total NK cells) in NHP DE3V. 

 In comparing the NK cell increases in the blood and the LNs, the magnitude of NK cell increases in the blood is not similarly observed in the LNs for a given NHP. For example, NHP AT25S in the blood had the highest NK cell number in the group (Figure 1A), but the LN NK cell number was relatively low compared to other NHPs (Figure S2). In contrast, NHP DE3V had the highest NK cell number in the LN, but the NK cell number in the blood in this NHP is relatively low (Figure S2 and Figure 1A).

### Robust NK cell proliferation in the blood and LNs following MPXV challenge

**Figure 3:**
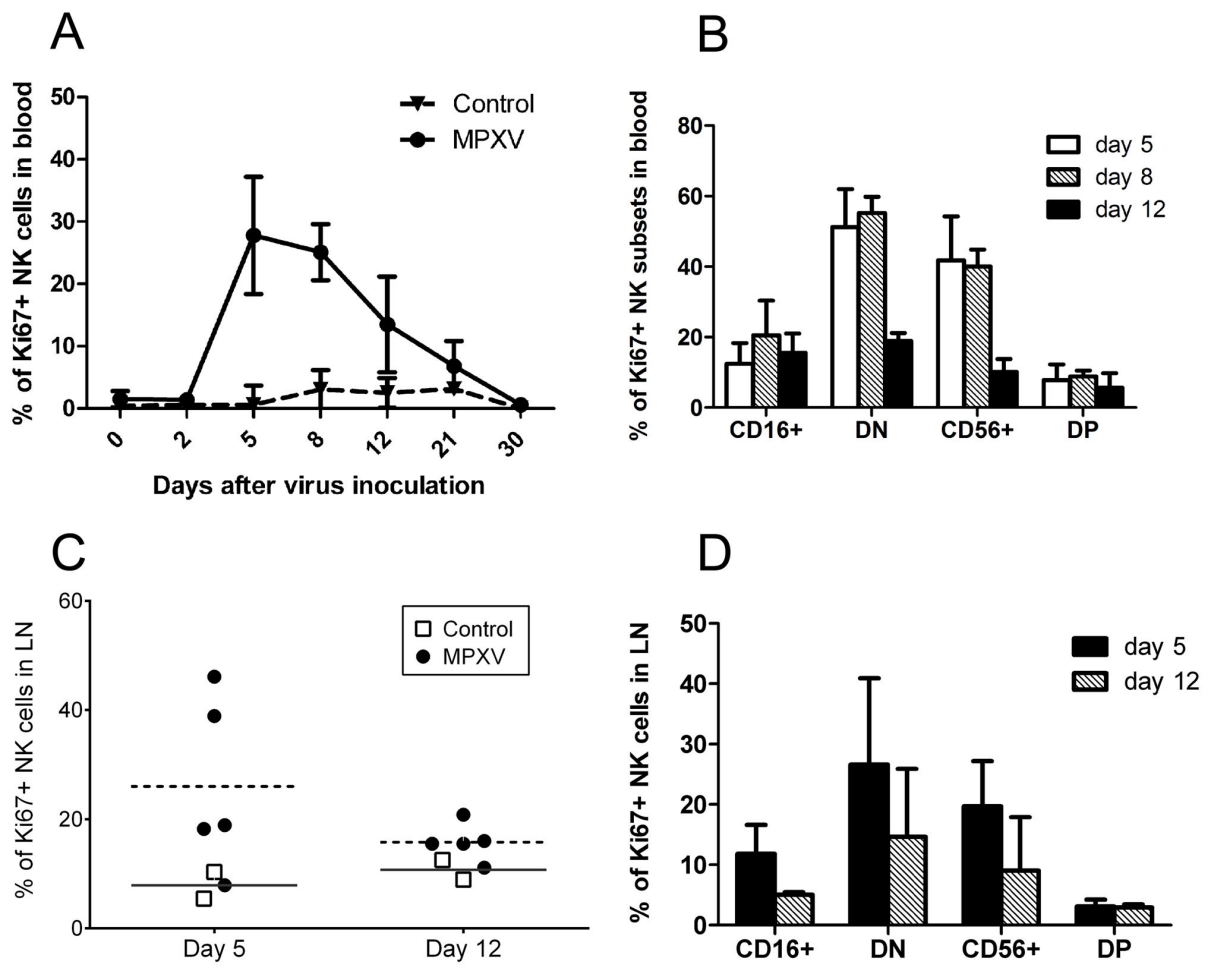
Proliferation of NK cells from blood and lymph nodes upon MPXV infection. A) Frequency of Ki67+ NK cells within the NKG2A+ NK cell gate. B) Frequency of Ki67+ NK cells in each NK subset from days 5, 8, and 12 blood samples. C) Ki67+ NK cell frequency within the NKG2A+ cell gate from days 5 and 12 biopsies of LNs. D) Ki67+ cell frequency in each NK subset from days 5 and 12 biopsies of LNs (bar represent the median frequency of Ki67+NK cells).

 The marked increase of NK cells in the blood and LNs could be largely due to cell proliferation. In a previous study in mice infected with either MCMV or VACV, peripheral NK cells rapidly proliferated and peaked at day 6 postinoculation [43]. Similarly, ECTV infection also induced NK proliferation in mice [20]. To assess NK cell proliferation in our study, we examined Ki67 expression in the NK cells from the blood and LNs. NK cells from the blood of normal control NHPs and NHPs before virus inoculation showed a low frequency of Ki67 staining (<5%), and the Ki67 expression frequency at day 2 after virus inoculation was similar to background (Figure 3A). However, at days 5–8 after MPXV inoculation, about 30% of NK cells in the blood expressed Ki67 protein (Figure 3A), a time frame consistent with previous reports [20,43] and with the NK cell number change in the blood (Figure 1A). The frequency of Ki67+ cells in the blood gradually reduced to baseline at days 21–30. When we examined the proliferation of NK cell subsets, CD16-negative cells in the blood including DN cells and CD56+ cells dominantly expressed Ki67 protein (Figure 3B). While a low frequency of CD16+ (~15-20%) and DP (~5-8%) populations also expressed Ki67, the Ki67+ cells in these two populations tended to express a low intensity of CD16 protein (data not shown). 

 We next measured Ki67 expression in NK cells in LNs at days 5 and 12 postinoculation and found that a higher proportion of LN NK cells from the MPXV-infected NHPs at day 5 expressed Ki67 (average 26%) compared to NK cells from control NHPs (average 7.9 %) (Figure 3C). In addition, day 5 LNs from infected NHPs seemed to have a higher frequency of Ki67+ cells than day 12, supporting the notion that cell proliferation peaked at an early stage of virus infection (Figure 3C, 3D) [20,43]. Similar to blood NK cells, LN CD16^-^ NK cells (DN and CD56+ NK cells) displayed higher Ki67 staining than CD16^+^ (CD16+ and DP) cells (Figure 3D). 

### Delayed/reduced chemokine receptor expression on NK cells following MPXV challenge

**Figure 4:**
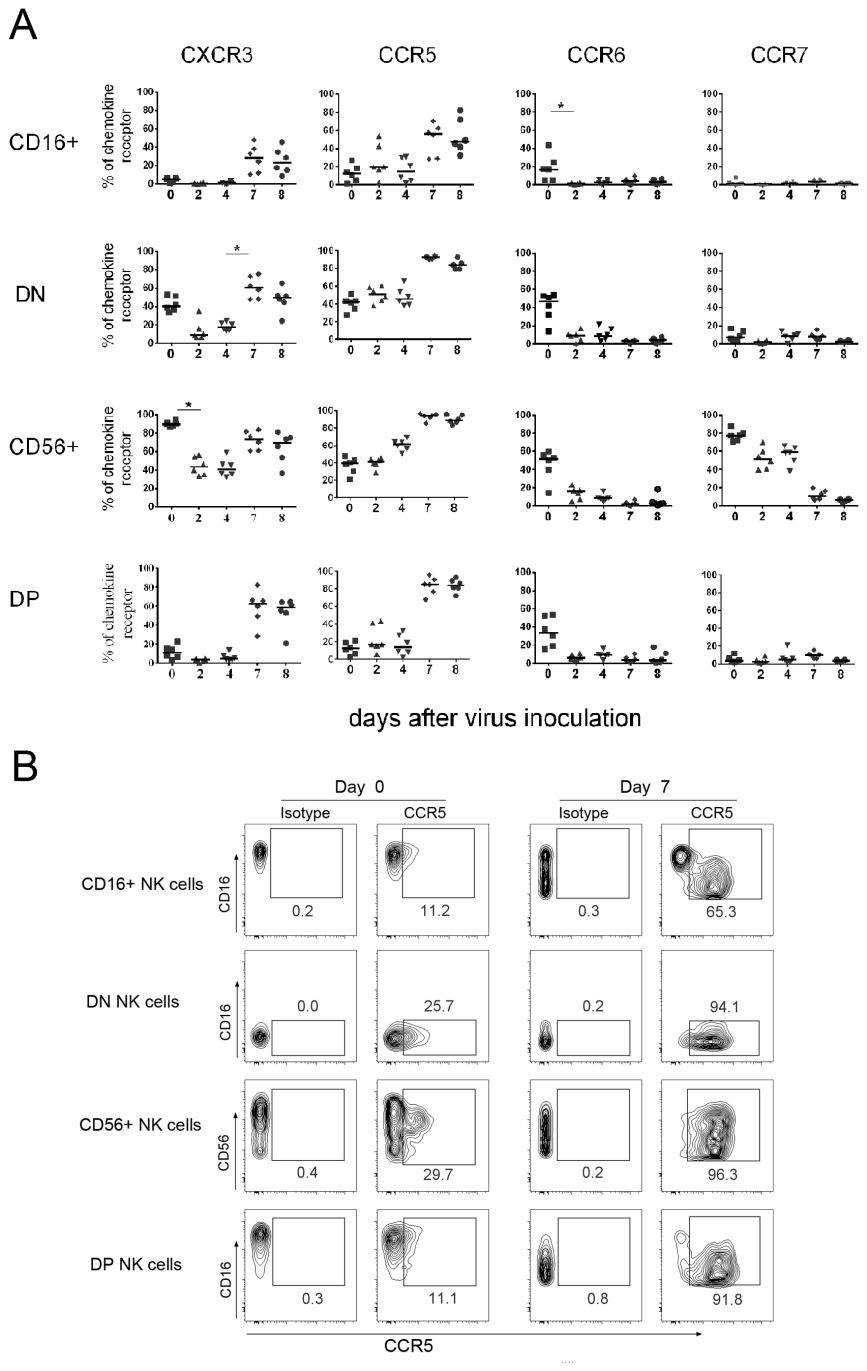
MPXV infection induced changes in the expression of chemokine receptors on blood NK cells. PBMCs from different time points following MPXV inoculation were stained for NK markers and chemokine receptors as described in the material and methods. The chemokine receptor expression was revealed by gating on individual NK cell subsets. A) Expression kinetics of CXCR3, CCR5, CCR6, and CCR7 on NK cell subsets following MPXV inoculation. B) Representative analysis of CCR5 expression on NK cells at days 0 and day 7 post-inoculation. Statistical analyses were permutation re-sampling tests with Holm-adjusted p-values computed using R (*p<0.05).

 To study the potential of NK cells to migrate to infected peripheral tissues upon MPXV infection, we assessed the expression of chemokine receptors CXCR3, CCR5, CCR6, and CCR7 on each NK cell subset. Similar to result from others [31,44], our analysis of NK cells from pre-inoculation blood showed that CD56+ cells (average 40%) and DN cells ( average 42%) were the major subsets expressing CCR5 (Figures 4A) . In contrast, only a very small proportion of CD16+ and DP NK cells express a low intensity of CCR5. The frequency of CCR5-expressing cells in all subsets remained unchanged at day 2 after MPXV infection and unchanged for CD16+, DN, and DP NK cells at day 4, but moderately increased in CD56+ cells (from average 40 to 60%, p>0.05) at day 4. At day 7, however the average frequency of CCR5-expressing cells in all subsets largely increased but did not attain a statistical significance (Figure 4A). In addition, the intensity of CCR5 expression also increased at day 7 after MPXV inoculation as shown in a representative NHP in Figure 4B. Interestingly, the CCR5-expressing cells in CD16+ and DP population were CD16^dim^ cells (low intensity of CD16 expression), suggesting the CD16^dim^ population might be preferentially recruited to tissues compared to CD16^high^ NK cells (Figure 4B).

 CCR7 is the chemokine receptor required for T and NK cells to home to LNs [45-47]. Similar to result from other reports [31,46], our results showed that only CD56+ NK cells at steady state expressed CCR7 (Figure 4A). Following MPXV infection, the frequency of CCR7+ CD56+ cells gradually decreased from 78% at day 0 to 50–60% on days 2–4 and to a low or negative frequency at days 7–8. MPXV infection did not induce a distinguishable change on CCR7 expression in other three NK populations that were negative at steady state and during MPXV infection (Figure 4A). 

 CXCR3 has been shown to mediate the migration of effector T cells and NK cells to lymphoid tissues and/or inflamed tissues [48,49]. Consistent with reports from others, we found that about 90% of circulating CD56+ NK cells and 40% of DN cells expressed CXCR3 at steady state, but CD16+ and DP cells did not express CXCR3 (Figure 4A). Following MPXV inoculation, the frequency of CD56+ cells and DN cells that expressed CXCR3 was first reduced (p<0.05 CD56+, p>0.05 DN) at days 2 and 4, and then largely recovered at days 7–8 (p<0.05 for DN cells). In addition, about 20-25% of CD16+ cells and 60% of DP NK cells also expressed CXCR3at days 7–8. 

The role of CCR6 in cell migration is less clear than the other chemokine receptors. Some investigators indicated that CCR6 may play a role in recruiting NK cells to tissues [31]. Also consistent with a previous report of SIV-infected macaques [31], results from our data showed that various proportions of NK subsets before virus inoculation express CCR6. MPXV infection ablated CCR6 expression on all four NK cell subsets (Figure 4A). 

**Figure 5:**
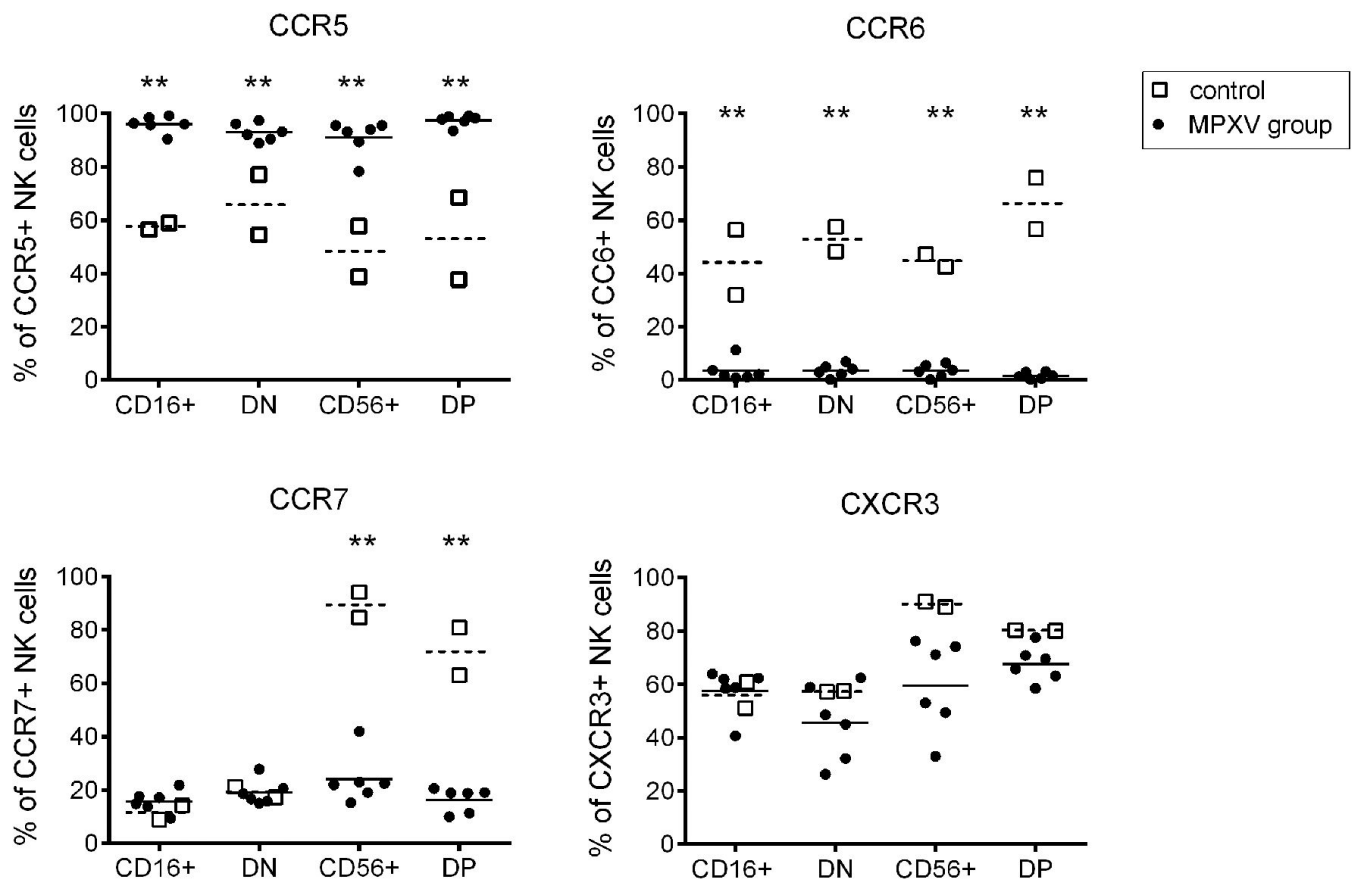
MPXV infection induced changes in chemokine receptor expression on NK cells in LNs. Frequency of chemokine receptor expression on NK cell subsets from the axillary LNs of day 8-9 MPXV-infected NHPs. Statistical analyses were permutation re-sampling tests with Bonferroni-adjusted p-values computed using R (**p<0.01).

We further analyzed chemokine receptor expression on NK cells in the axillary LNs from control NHPs and from NHPs at necropsy days 8–9 following MPXV inoculation. In control NHPs, about 45–80% of all four NK subsets from LNs expressed CCR5, CCR6, and CXCR3, and only CD56+ and DP cells expressed CCR7 (Figure 5). Upon MPXV infection, CCR5-expressing NK cells greatly increased (p<0.01) and almost all LN NK cells expressed CCR5 (Figure 5). LN CD16+ and DN NK cells did not express CCR7 at steady state and during MPXV infection. However, the CCR7-expressing cells in CD56+ and DP population were markedly reduced (p<0.01) upon MPXV infection, a result consistent with that from blood NK cells. The expression of CXCR3 did not change in CD16+ and DN cells during MPXV infection, but slightly decreased in CD56+ and DP cells (p>0.05). Similar to circulating NK cells, almost all NK cells in the LN lost CCR6 expression following MPXV infection. 

### Reduced CD107a expression and ablated cytokine secretion by NK cells

**Figure 6:**
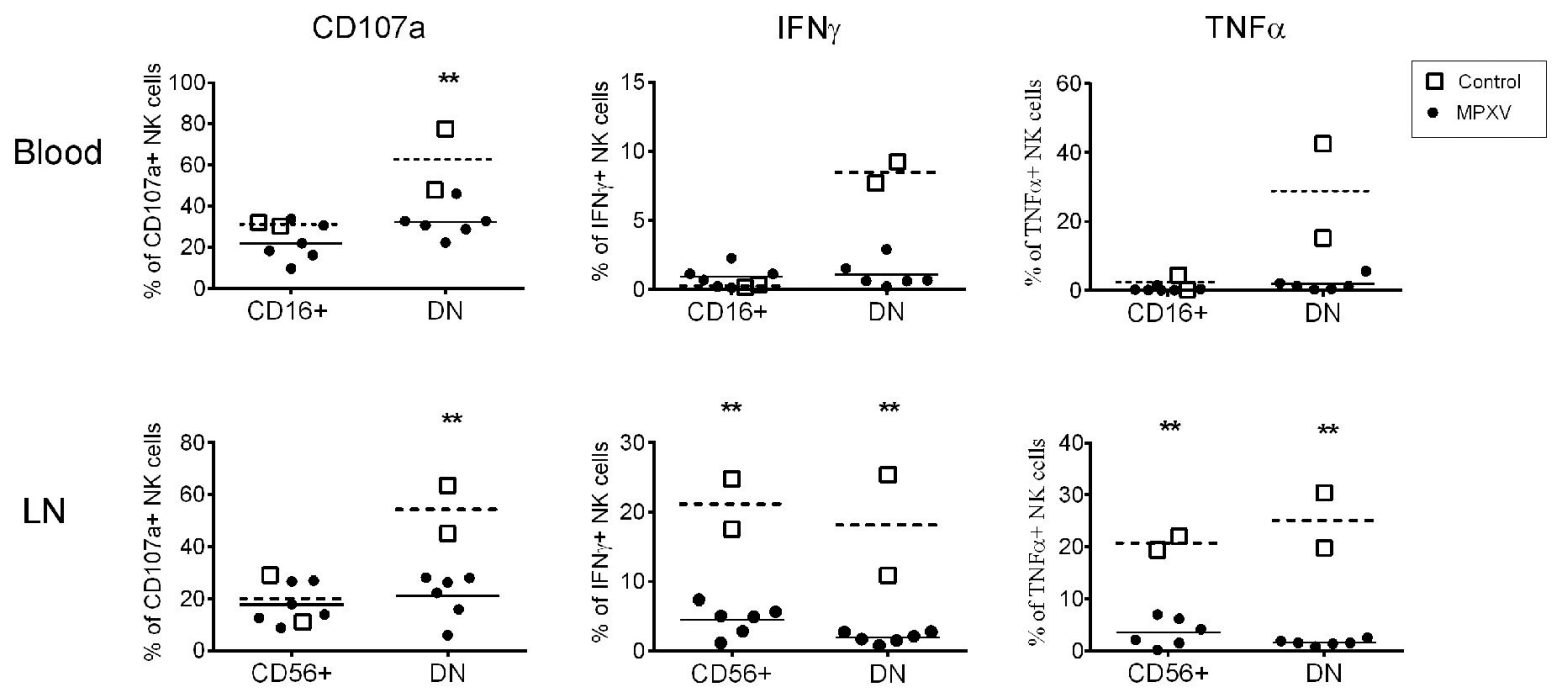
Compromised NK cell functions upon MPXV infection. Frequency of the expression of CD107a, IFN-γ, and TNF-α by different NK cell subsets from day 8-9 blood and LN samples is shown. Calculation of mean frequency of expression from control (dotted line) and MPXV-infected NHPs (solid line) was performed with Prism. Statistical analyses were permutation re-sampling tests with Bonferroni-adjusted P-values computed using R (**p<0.01).

 We next asked whether MPXV infection induces any changes in NK cell functions, including cytotoxicity capacity and secretion of cytokines. We assessed the expression of CD107a, a molecular marker for degranulation and cytotoxicity [50,51], and intracellular expression of IFN-γ and TNF-α. Enriched NK cells from blood and LNs were stimulated with MHC-I-devoid cell line 721.221, and the expression of surface CD107a and intracellular cytokines were measured. Consistent with published reports [31], circulating CD16^+^ NK cells from control NHPs expressed CD107a and barely expressed IFN-γ and TNF-α (Figure 6), supporting their major role in killing infected cells. DN cells, on the other hand, displayed strong expression for both CD107a and cytokines. It is believed that CD56+ NK cells are specialized to produce cytokines, such as IFN-γ and TNF-α [31,33] and our data from normal LN control NK cell cultures support the cytokine production role of CD56+ NK cells by showing that about 20% of CD56+ cells stained positive for IFN-γ and TNF-α (Figure 6). However, we also observed that 20% of CD56+ NK cells from the normal LN culture expressed CD107a. Similar to the NK cells in the blood, DN cells from the normal control LNs expressed both CD107a and cytokines. Upon MPXV infection, the frequency of CD107a-expressing CD16+ cells from the blood and the CD56+ cells from the LN remained unchanged. However, CD107a expression in the DN cells from both blood and LNs was significantly reduced (p<0.01). Most strikingly, almost all the NK cells, regardless of the source (blood and LNs) and subset, lost their capacity to express both IFN-γ and TNF-α, indicating a crippled NK cell cytokine response following MPXV infection. 

### Clinical signs, virus load, and circulating NK cell responses

 NHPs in both experiments A and B received a sub-lethal dose of MPXV and all NHPs survived through each individual study end point (Table 1). In general, all NHPs developed typical clinical signs of MPXV infection including fever, weight loss and skin lesions [4]. No apparent difference was observed regarding the clinical measurements (CBC/diff and serum chemistry) and clinical signs among the NHPs from both experiments. No clear correlation between the maximal lesion number and the peak NK cell number at day 7-8 was established (R=0.7 and *p=0.233*, data not shown). Virus load (viral DNA copy number measured by qPCR) was used in previous studies as a parameter to predict disease progression [52,53]. Our studies using qPCR test of whole blood samples showed the onset of positive MPXV DNA was from days 2-7, roughly paralleling the increases in NK cell number and lymphocyte number in the blood. Viremia lasted from a few days to 10-11 days (data not shown). The peak MPXV genome copies varied among individual NHPs and between the two experiments (Table 2). One out of six NHPs from experiment A and two out of four NHPs from experiment B were MPXV negative at all time-points. The sample from NHP DC98 was contaminated and thus not included in analysis. Statistical analysis showed no clear correlation between virus load and NK cell number (p>0.05, data not shown). 

**Table 2:** Peak virus load and peak NK cell number in the circulating blood.

Experiments	NHPs	Log_10_ peak viral genome copies/ml of blood	Day of peak virus load		Peak NK cell number/µl of blood	Day of peak NK cell number
	AT25S	OOR*	NA**		4946	7
	CG3J	4.82	9		1840	7
A	DE3V	5	8		2005	7
	HDX	4.65	7		3531	7
	iD3	4.35	7		1627	7
	XAV	4.07	7		2856	8
	A5E068	OOR	NA**		1563	8
	BDW5	6.86	12		1802	8
B	DC90	5.75	12		2897	8
	E06	OOR	NA**		1593	8

OOR, out of range low; ** NA, not available; NHP DC98 in experiment B was excluded for analysis

##  Discussion

 We report for the first time the kinetics of NK cell responses to MPXV infection in the rhesus macaque model. Previous study with ectromelia virus-infected mice showed only a modest increase in NK cell number (2–4 fold) following viral infection [22]. Our results with MPXV infection in NHPs showed a much greater increase in NK cell number both in the blood (23-fold) and in LNs (46.1-fold). In addition, MPXV induced a greater increase in NK number compared to that of the total lymphocytes (23-fold versus 3-fold). These data and the observation that lymphadenopathy is the hallmark of MPXV infection [54,55] prompt a question whether the robust NK cell proliferation and the marked increase in NK cell number induced by MPXV infection is unique to MPXV. It is possible that MPXV expresses specific proteins or peptides to either directly bind NK cell activating receptors to trigger activation signals or reduce inhibitory signals by altering/reducing the interaction of NK cell inhibitory receptors and their ligands such as by down-regulating MHC-I expression. Previous studies have shown that several viruses including ECTV and VACV express specific proteins to bind/modulate NK cell receptors and to induce NK cell proliferation and activation [8,18,22,56-58]. However, the difference in the magnitude of the NK cell response to challenge with MPXV and ECTV could also be due to differences in animal species studied (mouse versus rhesus macaques). Further experiments should compare the NK cell responses induced by MPXV to that induced by other poxviruses such as cowpox viruses and variola virus in NHPs.

 In our study, marked variation was observed in the changes in NK cell number both in the blood and in LNs among individual NHPs. However, when the overall changes in NK cell number both in the blood and LN in an individual NHP are compared to other NHPs, it is difficult to determine the individual NHP that had the strongest overall NK response. The different changes of NK cell number in the blood and LNs is possibly related to differential recruitment of NK cells from and to the lymphoid tissues or other tissues to or from the blood. If a true marked variation in NK cell response between NHPs exists, this variation could be largely due to difference in genetic polymorphism and polygenic of NK cell receptors [8,59,60]. In addition, individual immunological environment in the NHP such as different immune cell compositions and the baseline cytokine concentration may also play a role. 

 The marked increases of blood NK cells were manifested by increases of all four NK cell subsets with DN cells showing the highest increases. In addition, the composition of NK cell subsets in the circulating blood and in the LNs also largely altered. In addition to CD16+ subset, DN and CD56+ cells also become prominent populations in the blood. In the LNs, all four NK subsets co-exist. These data indicate a very dynamic/versatile NK cell response and probably overlapping roles among the different subsets of NK cells during MPXV infection. In future analysis, inclusion of multiple NK cell markers is necessary to provide a global picture of the response.

 Results from previous studies showed that CD56+ human NK cells proliferated more vigorously than CD16+ cells upon activation, and CD56+ NK cells can differentiate into CD16+ cytotoxic NK cells [61]. In our study, we showed that both CD56+ cells and DN cells from blood and LNs displayed the highest level of Ki67 expression, and CD16+ cells had little Ki67 expression. These data suggest that proliferating CD56+ cells may differentiate into transitional DN NK cells, then terminally into cytotoxic CD16+ cells. The increases in CD16+ NK cell number in the blood and LNs and the demonstration that DN cells are potent in both cytotoxicity and secretion of cytokines support this hypothesis. Therefore, we speculate that these transitional DN cells are an important cell population that exerts multiple NK cell functions.

 To exert their antiviral functions, blood NK cells need to be recruited to infected tissues, a process mediated by chemokine receptors, chemokines, and adhesion molecules. Except for an increase in CCR5 expression at day 4 in CD56+ blood NK cells, most blood NK cell subpopulations had a reduced/low frequency of chemokine receptor expression during early MPXV infection. The low number of some blood NK subsets (DN, CD56+, DP NK cells) and the generally low and/or reduced frequency of expression of chemokine receptors on these cells during early MPXV infection indicate that NK cells have a very limited capacity to migrate to peripheral tissues. 

At days 7/8 post-inoculation, the expression of CXCR3 and CCR5 was recovered and/or increased on majority of blood NK cells. In contrast, almost all NK cells lost their expression of CCR7 and CCR6, indicating that CXCR3 and CCR5 mediated the migration of NK cells into peripheral tissues. Our data are consistent with a previous study that showed NK cell entry into stimulated LNs is mediated by CXCR3 rather than by CCR7, although recruitment of NK cells under normal conditions depends on CCR7 [13]. In addition, our data also support previous conclusion that CCR5 is a critical chemokine receptor, guiding NK cell trafficking to infected tissues where NK cells induce inflammatory responses, kill infected cells, and modulate adaptive immune responses [62-64] 

Several caveats of our study include lack of assessment of the expression of other important chemokine receptors that also mediate NK cell recruitment, such as CX3CR1 on CD16+ NK cells [44,65] and the analysis of expression gradients of chemokines in individual tissues. Lack of these data limits us from providing a precise picture of the kinetics of NK cell migration. Therefore, we conclude that our chemokine receptor analysis only indicates the potential of NK cells to migrate to peripheral tissues at day 7/8 following MPXV infection. 

 Assuming NK cells successfully migrated into the inflamed tissues at day 7/8 following MPXV infection, do they actually exert their supposed functions? Our results showed that the major NK cell population in the blood at day 7/8, DN cells, had a reduced CD107a expression, an indication of a compromised cytotoxicity. More strikingly, almost all NK cells lost their cytokine secretion capacity. This result is in stark contrast to results from the study with ECTV in C57BL/6 mice where ECTV infection induced both NK cell activation, cell proliferation, IFN-γ secretion, and cytotoxicity [22]. The ablation of cytokine expression by MPXV infection could have serious consequences. First, compromised IFN-γ production may directly reduce the capacity of NK cells to clear MPXV-infected cells as both IFN-γ and perforin are required to clear ECTV [22]. Secondly, full battle of the immune system against virus infection requires the collaboration of different arms of immunity such as T cell responses and virus-specific Abs to handle large virus loads. NK cells play a key role in initiating and shaping adaptive immune response, especially T cell responses by collaborating with DCs and by secreting cytokines [13]. One can speculate that the deficiency of cytokine secretion by NK cells could result in delayed/reduced T cell activation and the following B cell activation [66-68]. 

 All NHPs in our experiments were treated with a sub-lethal dose of virus and survived through the study endpoints. All NHPs developed typical clinical signs of MPXV infection and no clear difference on the clinical signs was observed among the NHPs. However, three out of ten NHPs had no detective virus DNA and the NHPs with positive MPXV DNA showed marked variation in the onset, duration and magnitude of viral genome copies, making it difficult to establish a clear correlation between virus load and NK cell responses statistically. Although the potential of NK cell migration at early time points was reduced and NK cells lost their cytokine secreting activity, the large increase of NK cell number, the recovery of chemokine receptor expression at day 7/8 and the relatively normal or partially reduced CD107a expression on NK cells indicates that NK cells preserved, at least partially, their function such as killing capacity, thus may contributing to the final virus clearance. However, this NK cell killing effect may be limited when lethal doses of virus are injected. From our studies, we cannot rule out the possibility that other factors also play important role in limiting virus replication and virus load. Those factors may explain why we observed marked variation in virus load and the virus load did not correlate with NK cell response.

 In summary, our data depict a complex scenario on the evolution of MPXV-induced NK cell response. NK cell responses to MPXV infection are controlled at multiple levels such as increased NK cell number in the blood and in the lymphoid tissues, capacity to be recruited to infected tissues, and NK cell cytotoxicity and secretion of cytokines. Our data suggest reduced migration of NK cells to tissues at early stage of MPXV infection. We also found a dynamic change in the expression of chemokine receptors on NK cells during MPXV infection. At resting stage, CCR7 mediates the recruitment and retention of NK cells in the LNs [42]. However, CCR5 and CXCR3 could be the major receptors mediating recruitment of NK cells to lymphoid tissues during MPXV infection. MPXV infection also induced reduction of NK cell cytotoxicity and ablated NK function to secrete cytokines. Taken together, although MPXV induced massive NK cell proliferation and increases in NK cell number, the cell migration and function were reduced or even completely ablated. This may be a causal reason why most NHPs succumb to MPXV infection following high doses of MPXV infection. 

## Supporting Information

Figure S1 **MPXV infection induced increases in total lymphocyte number in the blood.** The absolute number of total lymphocytes in the blood from experiment A (A) and experiment B (B) were determined with BD Trucount tubes. Total lymphocyte was gated based on Forward scatter and CD45 expression.
(TIF)

Figure S2 **NKG2A+ NK cell numbers in LNs of individual NHPs at day 8-9 post MPXV inoculation.** Total NK cell number was calculated based on the frequency of NKG2A+ NK cells in the lymphocyte gate and the total live cell number of individual LNs.
(TIF)
