# Supplementary figures and images for: Monkeypox Virus Infection of Rhesus Macaques Induces Massive Expansion of Natural Killer Cells but Suppresses Natural Killer Cell Functions
Source: PLoS One. 2013 Oct 17;8(10):e77804. doi: 10.1371/journal.pone.0077804 (PMC3798392; doi:10.1371/journal.pone.0077804)

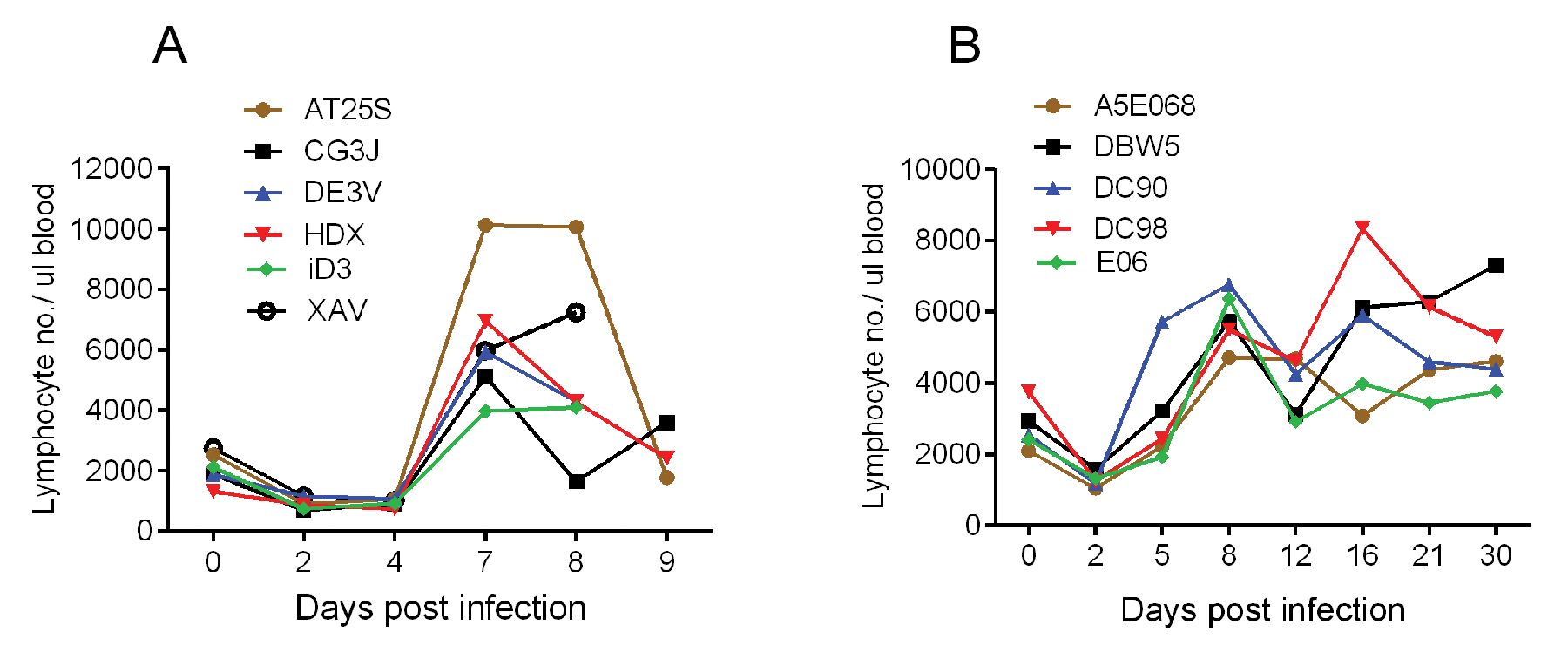

Supplement: Figure S1 — MPXV infection induced increases in total lymphocyte number in the blood. The absolute number of total lymphocytes in the blood from experiment A (A) and experiment B (B) were determined with BD Trucount tubes. Total lymphocyte was gated based on Forward scatter and CD45 expression. (TIF) [file pone.0077804.s001.tif]

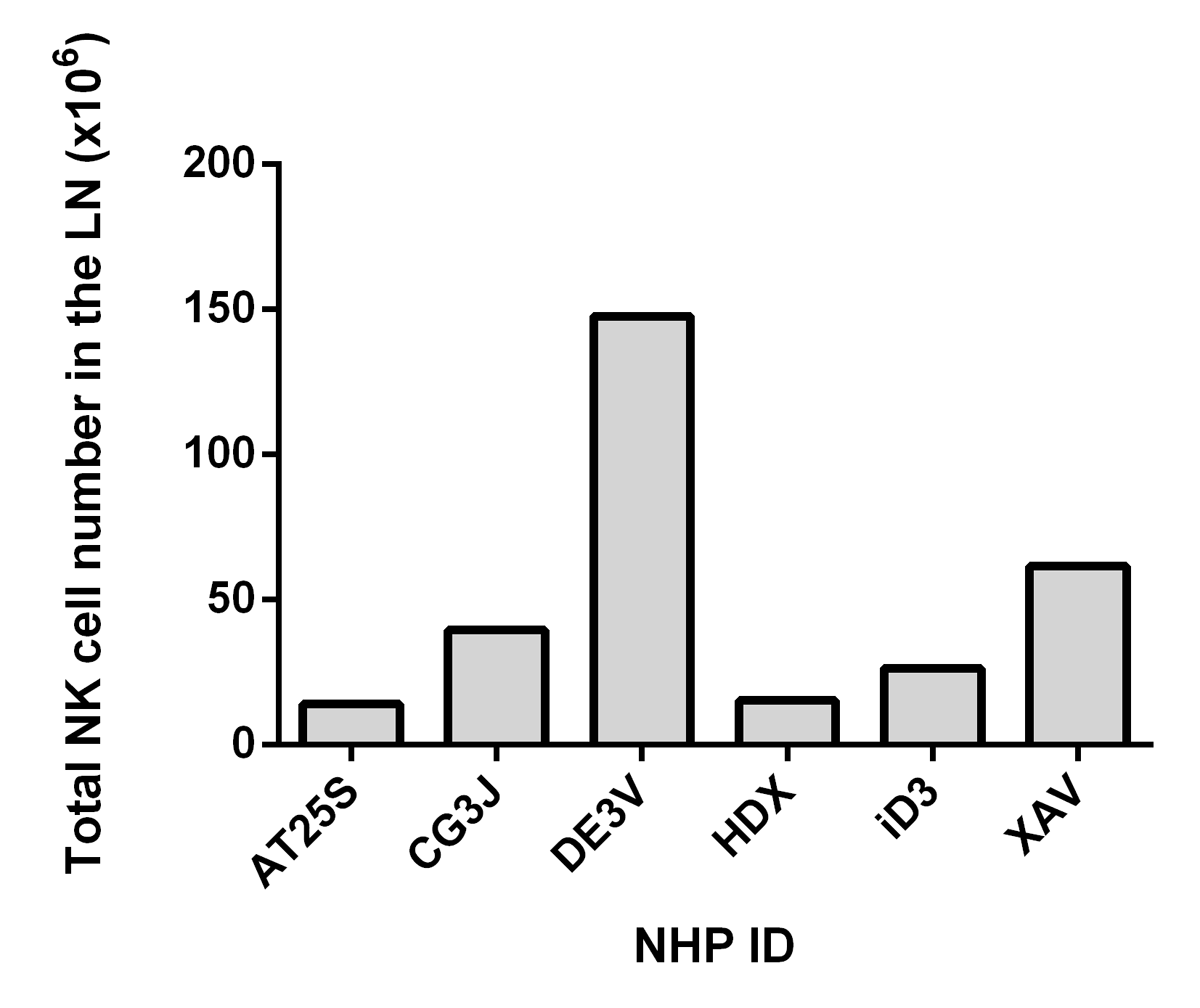

Supplement: Figure S2 — NKG2A+ NK cell numbers in LNs of individual NHPs at day 8-9 post MPXV inoculation. Total NK cell number was calculated based on the frequency of NKG2A+ NK cells in the lymphocyte gate and the total live cell number of individual LNs. (TIF) [file pone.0077804.s002.tif]
